# Coronary Endothelium No-Reflow Injury Is Associated with ROS-Modified Mitochondrial Fission through the JNK-Drp1 Signaling Pathway

**DOI:** 10.1155/2021/6699516

**Published:** 2021-01-30

**Authors:** Yi Chen, Chen Liu, Peng Zhou, Jiannan Li, Xiaoxiao Zhao, Ying Wang, Runzhen Chen, Li Song, Hanjun Zhao, Hongbing Yan

**Affiliations:** ^1^Department of Cardiology, Fuwai Hospital, National Center for Cardiovascular Diseases, Peking Union Medical College, Chinese Academy of Medical Sciences, Beijing, China; ^2^Fuwai Hospital, Chinese Academy of Medical Sciences, Shenzhen, China

## Abstract

Coronary artery no-reflow is a complex problem in the area of reperfusion therapy, and the molecular mechanisms underlying coronary artery no-reflow injury have not been fully elucidated. In the present study, we explored whether oxidative stress caused damage to coronary endothelial cells by inducing mitochondrial fission and activating the JNK pathway. The hypoxia/reoxygenation (H/R) model was induced *in vitro* to mimic coronary endothelial no-reflow injury, and mitochondrial fission, mitochondrial function, and endothelial cell viability were analyzed using western blotting, quantitative polymerase chain reaction (qPCR), enzyme-linked immunosorbent assay (ELISA), and immunofluorescence. Our data indicated that reactive oxygen species (ROS) were significantly induced upon H/R injury, and this was followed by decreased endothelial cell viability. Mitochondrial fission was induced and mitochondrial bioenergetics were impaired in cardiac endothelial cells after H/R injury. Neutralization of ROS reduced mitochondrial fission and protected mitochondrial function against H/R injury. Our results also demonstrated that ROS stimulated mitochondrial fission via JNK-mediated Drp1 phosphorylation. These findings indicate that the ROS-JNK-Drp1 signaling pathway may be one of the molecular mechanisms underlying endothelial cell damage during H/R injury. Novel treatments for coronary no-reflow injury may involve targeting mitochondrial fission and the JNK-Drp1 signaling pathway.

## 1. Introduction

Primary percutaneous coronary intervention (PPCI) is the gold standard for the treatment of ST segment elevation myocardial infarction (STEMI) because it results in the timely opening of the occluded coronary arteries [1]. Fresh blood flow is restored in >90% of STEMI patients after PPCI. However, there is still a small number of patients who develop severe microvascular obstruction (MVO) even though epicardial arteries are successfully opened following PPCI. This phenomenon is called coronary no-reflow injury [2]. The early clinical manifestation of patients with coronary no-reflow injury includes recurring angina pectoris, malignant arrhythmias (e.g., ventricular tachycardia, ventricular fibrillation, and atrioventricular block), acute left heart failure, cardiogenic shock, and cardiac rupture or other malignant complications [3]. Although the incidence of coronary no-reflow varies between studies because of differences in evaluation methods and types of no-reflow injuries used [4], it is estimated that the incidence of no-reflow injury is ~26% in patients with anterior wall STEMI [5].

It is believed that the initial cause of coronary no-reflow injury is related to microvascular dysfunction [6]. The myocardial blood supply is composed of the subepicardial coronary artery and the capillaries that penetrate the myocardium [7], and microvessels form the main resistance to myocardial perfusion blood flow [8]. Further, coronary blood flow is inversely correlated with microvascular resistance. Several mechanisms, including endothelial cell death, inflammation response, oxidative stress, and coronary spasm, have been introduced to explain microvascular damage after PPCI [9–12]. It was reported that the microvessel wall was thickened, the lumen became smaller, and the endothelial cells were swelling in patients undergoing myocardial biopsy [13]. It has also been reported that endothelial cell damage is primarily caused by oxidative stress; however, the detailed molecular mechanisms underlying this damage have not been fully elucidated [14].

Although previous studies have reported that excessive oxidative stress is associated with mitochondrial dysfunction [15] (as evidenced by decreased mitochondrial membrane potential and increased mitochondrial proapoptotic factor release), the mechanisms underlying ROS-induced mitochondrial damage in endothelial cells following coronary no-reflow injury remain unknown. Recent investigations have demonstrated that mitochondrial dynamics disorder may be an upstream regulator of mitochondrial function [16–18]. Excessive mitochondrial fission divides mitochondria into several fragmentations that contain damaged mitochondrial DNA [19]. Since these fragmented mitochondria cannot synthesize the normal mitochondrial respiration complex, the physiological mitochondrial membrane potential cannot be generated [20]. Additionally, a decreased mitochondrial membrane potential is associated with increased mitochondrial membrane hyperpermeability, which results in the leakage of proapoptotic proteins into the cytoplasm [21]. Interestingly, the relationship between ROS and mitochondrial dynamics disorders has not been fully elucidated [22]. A recent study illustrated that mitochondrial fission was mainly regulated by Drp1, which was activated through posttranscriptional phosphorylation following activation of the JNK pathway [23]. In the present study, we investigated whether ROS mediated endothelial cell damage by inducing mitochondrial fission via the JNK pathway. Specifically, the purpose of this study was to investigate the role of ROS-modified mitochondrial fission through the JNK-Drp1 pathway in cultured endothelial cells using a hypoxia/reoxygenation (H/R) injury model.

## 2. Materials and Methods

### 2.1. Cardiac Endothelial Cells (CECs)

Murine CECs were purchased from CELLutions Biosystems Inc. (Ontario, Canada) and cultured according to the supplier's instructions [24]. CECs were maintained in Dulbecco's Modified Eagle's Medium (glucose [4500 mg/L], L-glutamine [0.584 g/L], sodium bicarbonate [3.7 g/L], without sodium pyruvate [Sigma-Aldrich]) that was supplemented with penicillin (100 U/mL) and streptomycin (100 *μ*g/mL; both from GIBCO), 10 mmol/L HEPES (HyClone, Thermo Fisher Scientific), and 5% fetal bovine serum (FBS; Sigma-Aldrich). When confluent (2-3 days after plating), the CECs were harvested using a 0.25% trypsin/EDTA solution (GIBCO), subcultured, or used for functional assays and RNA purification [25].

### 2.2. ELISA

The contents of SOD, GSH, GPX, and MDA in the culture media were detected using ELISA kits following the manufacturer's instructions (Shanghai Hengyuan Biological Technology, China) [26]. The culture media was added to a microdroplet plate that was coated with purified SOD, GSH, GPX, and MDA primary antibodies and incubated at 37°C for 2 hours [27]. After incubation, the plate was washed and HRP-labeled secondary antibodies were added. After incubation with the secondary antibodies, the plate was washed thoroughly, and a solution of 3,3′,5,5′-tetramethylbenzidine substrate was added. A sulfuric acid solution was added to stop the reaction, and the color was analyzed using spectrophotometry at a wavelength of 450 nm [28]. The concentrations of SOD, GPX, GSH, and MDA in the culture media were then calculated according to the standard curve [29].

### 2.3. Immunofluorescence Analysis

The protocol for the immunofluorescent staining of the heart tissues was performed as described below. Samples were fixed using 4% paraformaldehyde and mounted on microscope slides [30]. The slides were subsequently treated with primary antibodies, and the relative immunofluorescence intensity was calculated as previously described [31].

### 2.4. Oxygen-Glucose Deprivation (OGD) Followed by Reoxygenation

Cells were incubated in 6-well plates (4 × 10^5^ cell/well) for 24 h, cultured in glucose-free DMEM, and incubated in an anaerobic chamber containing 95% N_2_ and 5% CO_2_ at 37°C for 3 h to mimic ischemic-like conditions *in vitro* [32]. After incubation, the CECs were returned to the atmosphere with 95% air and 5% CO_2_ and cultured in glucose-containing DMEM for 3 h for reoxygenation. The subsequent experiment was performed 24 h after reoxygenation [33].

### 2.5. RT-qPCR

Total RNA was extracted using TRIzol reagent kits (Invitrogen, CA, USA). After extraction, 1 *μ*g of total RNA was reverse transcribed to cDNA using the PrimeScript RT reagent kits (Takara) [34]. RT-qPCR was performed using a SYBR Premix Ex Taq II (TaKaRa) and the ABI 7500 real-time PCR system (Applied Biosystems). Relative RNA expression was normalized to that of GAPDH and was determined using the 2^−*ΔΔ*Ct^ method [35].

### 2.6. CCK-8 Assay

Transfected cells were seeded into 96-well plates and cultured for 24 h. Next, 10 *μ*L of the CCK-8 solution was added to each well, and the cells were incubated for 2 h. After incubation, absorbance was measured at 450 nm using a microplate reader (Bio-Rad, USA) [36].

### 2.7. Western Blot

Western blot was conducted as previously reported. The primary antibodies were purchased from Abcam (Cambridge, MA, USA), and HRP-conjugated secondary antibodies were obtained from Sangon (Shanghai, China) [37].

### 2.8. Mitochondrial Respiratory Chain Complex Activity Analysis

The activity of the mitochondrial respiratory chain was determined using the Mitochondrial Respiratory Chain Complex Activity Assay Kit (Solarbio, Beijing, China) according to the manufacturer's instructions [38]. Briefly, the mitochondrial complex was extracted from the ovaries using the suitable reagents, and 10 *μ*L of the extract was added to each well of a 96-well plate. Then, the detection reagents were added to the wells, mixed gently, and incubated at 37°C for 2 min. The corresponding absorbance was evaluated before and after the reaction using a microplate reader (BioTek, Vermont, VT), and the difference in absorbance was calculated [39]. Finally, the enzymatic activity of the complex was calculated using the corresponding formula that was provided in the kit manual [40].

### 2.9. Measurement of ATP Levels

ATP production was detected using the luminometric ATP Assay Kit (AAT Bioquest, Sunnyvale, CA) according to the manufacturer's instructions [41]. Briefly, 50 oocytes were seeded into each well of a 96-well white plate, and 200 *μ*L of the ATP assay solution was added to the oocyte cultures. After gently mixing and incubating for 20 min at room temperature [42], the luminescence intensity was measured using the luminometer mode on a plate reader (Tecan, Zurich, Switzerland). The readings were normalized to the total protein content [43].

### 2.10. Measurement of ROS Production

Cells were cultured in clear bottom 6-well black plates (Corning, Corning, NY, USA), incubated in the presence or absence of compounds for 48 h at 37°C, washed with PBS (pH 7.4), and incubated with 2′,7′-dichlorofluorescin diacetate (H2DCFDA) for 18 h [44]. Following incubation, the cells were washed twice with PBS (pH 7.4), and the fluorescence of 2′,7′-dichlorofluorescin (DCF) was then measured at excitation/emission (Ex/Em) wavelengths of 485/530 nm [45].

### 2.11. Statistical Analysis

Data shown are mean ± SEM of the number of independent experiments indicated (*n*) and represent experiments performed on at least three separate occasions with similar outcomes. Data were analyzed using one-way or two-way ANOVAs, and comparisons between groups were performed using a protected Tukey's test. Statistical significance was defined as *p* < 0.05.

## 3. Results

### 3.1. H/R Injury Mediates Endothelial Cell Oxidative Stress

**Figure 1:**
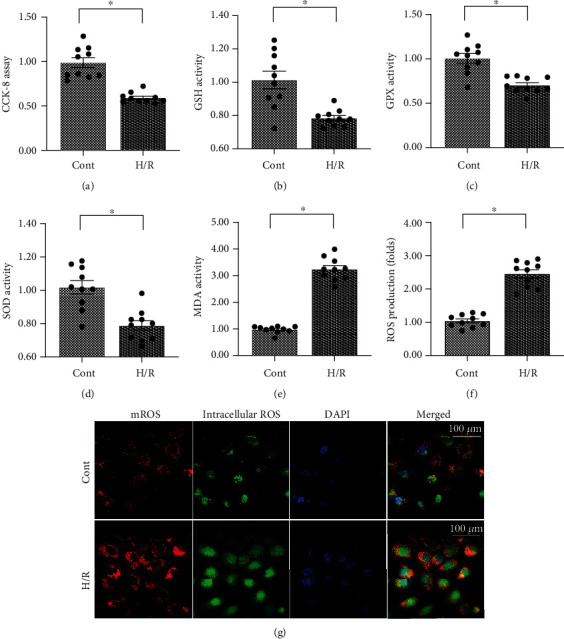
Hypoxia/reoxygenation injury mediates endothelial cell oxidative stress. (a) Cell viability was measured using the CCK-8 assay. Cardiac endothelial cells (CECs) underwent hypoxia/reoxygenation (H/R) injury. (b–e) The content of antioxidative factors was measured using an enzyme-linked immunosorbent assay (ELISA). (f, g) Intracellular ROS and mitochondrial ROS (mROS) were detected using DCFHDA and MitoSOX red mitochondrial superoxide indicator, respectively. ^∗^*p* < 0.05.

In the present study, an H/R model was used in cardiac endothelial cells (CECs) to mimic coronary microvascular no-reflow injury *in vitro*. Cell viability was measured using the Cell Counting Kit-8 (CCK-8) assay. As shown in Figure 1(a), cell viability was significantly suppressed in H/R-treated CECs as compared with control CECs. Recent studies have reported that oxidative stress is the primary pathological factor that induces endothelial cell damage during coronary no-reflow injury. To confirm this finding, an enzyme-linked immunosorbent assay (ELISA) was used to analyze alterations in the activities of antioxidants in CECs following H/R injury. As shown in Figures 1(b)–1(d), the activities of glutathione (GSH), superoxide dismutase (SOD), and glutathione peroxidase (GPX) were significantly increased in the H/R-treated cells as compared with the control group. This suggests that there was a decline in the antioxidative capacity of CECs following H/R injury. Additionally, the levels of malondialdehyde (MDA) were increased, and this may have been due to increased ROS production in the cytoplasm (Figure 1(e)). To further observe the oxidative stress in CECs, an ROS probe was used to stain the intracellular ROS. As shown in Figures 1(f) and 1(g), the levels of cytoplasmic ROS and mitochondrial ROS were significantly elevated in the H/R-treated CECs as compared with the control group. These results indicate that H/R is followed by oxidative stress in CECs under H/R injury.

### 3.2. Mitochondrial ROS Promotes Drp1 Phosphorylation and Mitochondrial Fission in Endothelial Cells following H/R Injury

**Figure 2:**
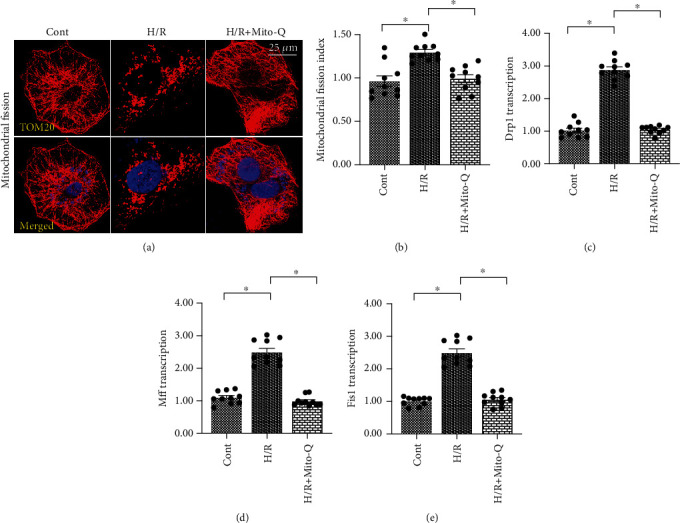
Mitochondrial ROS promotes Drp1 phosphorylation and mitochondrial fission in endothelial cells under hypoxia/reoxygenation injury. (a, b) Mitochondrial fission was measured using immunofluorescence. Cardiac endothelial cells (CECs) underwent hypoxia/reoxygenation (H/R) injury. Mito-Q, a mitochondrial antioxidant, was added to the CEC media to neutralize mROS. (c–e) qPCR was performed to analyze the transcription of Drp1, Mff, and Fis1 in CECs under H/R injury. ^∗^*p* < 0.05.

Previous studies have identified that mitochondrial ROS induces endothelial cell dysfunction through the disruption of mitochondrial homeostasis. Further investigations demonstrated that mitochondrial performance (especially mitochondrial fission) in endothelial cells is drastically regulated by mitochondrial dynamics. Therefore, we investigated whether mitochondrial ROS affected mitochondrial dysfunction in endothelial cells under H/R injury via activating mitochondrial fission. Mitochondrial morphology was observed through immunofluorescence. The data shown in Figures 2(a) and 2(b) demonstrated that the morphology of mitochondria was converted into a fragmented shape after exposure to H/R injury. To determine if mitochondrial ROS play a causal role in inducing mitochondrial fission, Mito-Q, a mitochondria-specific antioxidant, was incubated with CECs before H/R injury, and mitochondrial morphology was observed again after H/R injury. As shown in Figures 2(a) and 2(b), the elongated mitochondrial morphology was sustained in CECs that underwent Mito-Q treatment before H/R injury as compared with CECs that underwent H/R injury but were not treated with Mito-Q. To further support the role of mitochondrial ROS-induced mitochondrial fission, RNA transcriptions of mitochondrial fission-related factors were conducted. The transcriptions of Drp1, Mff, and Fis1 were significantly elevated in CECs that underwent H/R injury as compared with those in control CECs (Figures 2(c)–2(e)). Conversely, treatment with Mito-Q prevented the increase of these profission factors (Figures 2(c)–2(e)), suggesting that mitochondrial ROS plays a contributory role in initiating mitochondrial fission in endothelial cells.

### 3.3. Mitochondrial Dysfunction Is Induced by Mitochondrial ROS

**Figure 3:**
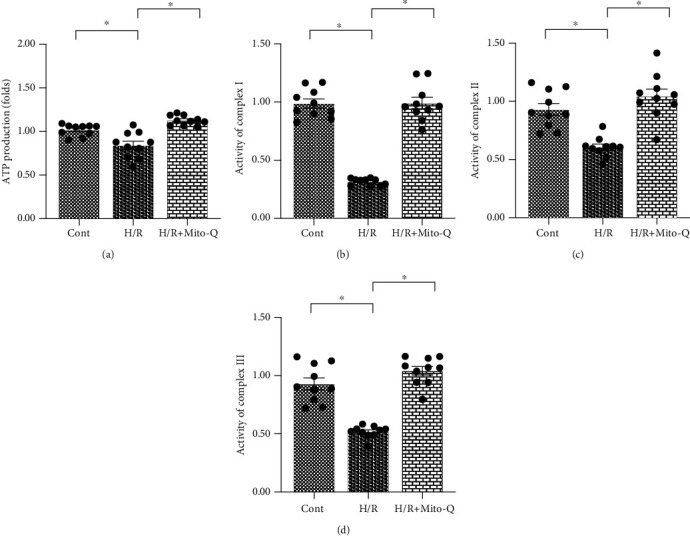
Mitochondrial dysfunction is induced by mitochondrial ROS. (a) Adenosine triphosphate (ATP) production was measured using an enzyme-linked immunosorbent assay (ELISA). Cardiac endothelial cells (CECs) underwent hypoxia/reoxygenation (H/R) injury. Mito-Q, a mitochondrial antioxidant, was added to the CEC media to neutralize mROS. (b–d) The activity of the mitochondrial respiration complex was determined using an ELISA. ^∗^*p* < 0.05.

Although we reported that mitochondrial fission could be activated by mitochondrial ROS in endothelial cells under H/R injury, it remains unclear whether mitochondrial function is disturbed by mitochondrial ROS in H/R-treated CECs. To address this question, mitochondrial function was assessed in response to Mito-Q treatment. As shown in Figure 3(a), cellular adenosine triphosphate (ATP) production, which is primarily generated by mitochondria, was downregulated in H/R-treated CECs as compared with control CECs. Additionally, ATP production was reversed to near-normal levels with Mito-Q administration. We also found that the activities of the mitochondrial respiration complex were reduced in H/R-treated CECs (Figures 3(b)–3(d)), and this alteration was improved by Mito-Q treatment. This finding suggests that mitochondrial ROS regulate mitochondria-mediated bioenergetics.

### 3.4. Inhibition of Mitochondrial Fission Also Sustains Mitochondrial Function in Endothelial Cells

**Figure 4:**
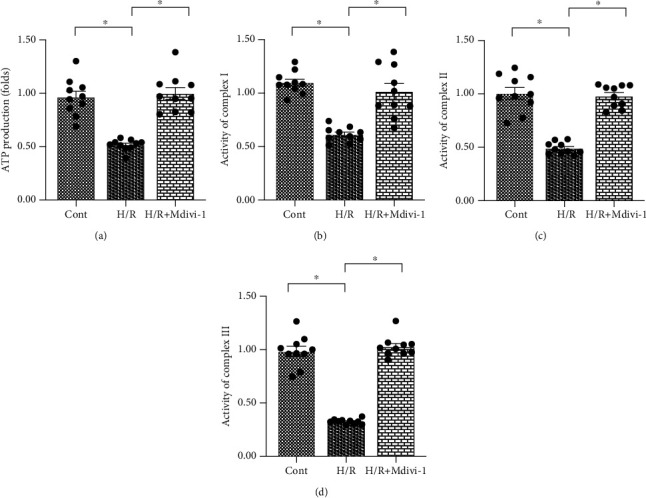
Inhibition of mitochondrial fission also sustains mitochondrial function in endothelial cells. (a) Adenosine triphosphate (ATP) production was measured using an enzyme-linked immunosorbent assay (ELISA). Cardiac endothelial cells (CECs) underwent hypoxia/reoxygenation (H/R) injury. Mdivi-1, a mitochondrial fission inhibitor, was added to the CEC culture media to repress mitochondrial fission. (b–d) The activity of the mitochondrial respiration complex was determined using an ELISA. ^∗^*p* < 0.05.

To understand whether mitochondrial fission is also involved in the regulation of mitochondrial function in endothelial cells under H/R injury, Mdivi-1, an inhibitor of mitochondrial fission, was added to the endothelial cell culture media, and mitochondrial function was evaluated again [46]. As shown in Figure 4(a), cellular ATP production was significantly reduced in H/R-treated endothelial cells as compared with control endothelial cells. Interestingly, administration of Mdivi-1 drastically improved ATP content in endothelial cells, and this was similar to the results obtained from the Mito-Q-treated endothelial cells. Our data also demonstrated that the activities of the mitochondrial respiration complex were inhibited in H/R-treated CECs (Figures 4(b)–4(d)). Interestingly, Mdivi-1 supplementation enhanced the activities of the mitochondrial respiration complex (Figures 4(b)–4(d)). These results suggest that mitochondria-related bioenergetics are normalized by mitochondrial fission inhibition.

### 3.5. ROS Causes Drp1-Related Mitochondrial Fission through the JNK Pathway

**Figure 5:**
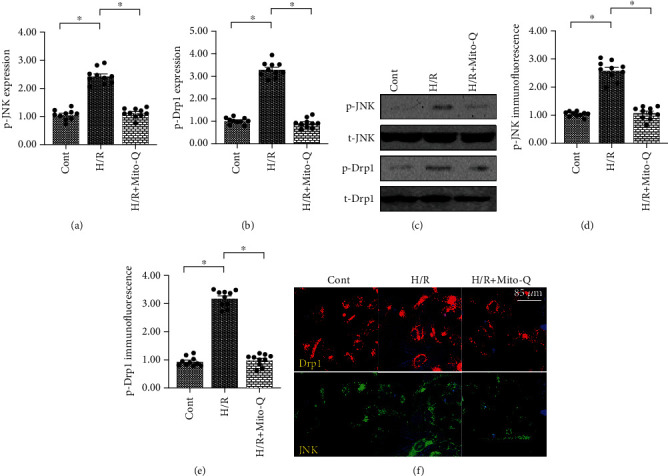
ROS causes Drp1-related mitochondrial fission through the JNK pathway. (a–c) Proteins were isolated from hypoxia/reoxygenation- (H/R-) treated CECs. Western blotting was performed to analyze the expression of JNK and Drp1. Mito-Q, a mitochondrial antioxidant, was added to the CEC culture media to neutralize mROS. (d–f) Immunofluorescence was performed to verify the expressions of JNK and Drp1. Mito-Q, a mitochondrial antioxidant, was added to the CEC culture media to neutralize mROS. ^∗^*p* < 0.05.

Lastly, we evaluated the molecular mechanism underlying ROS-induced mitochondrial fission. Previous studies have demonstrated that mitochondrial fission is mainly regulated by Drp1 phosphorylation, and Drp1 posttranscriptional phosphorylation can be modified by the JNK pathway [47]. Thus, we investigated if ROS-mediated mitochondrial fission could be achieved through JNK-induced Drp1 phosphorylation. Results from western blotting demonstrated that the JNK pathway was significantly activated by H/R injury in CECs as evidenced by the presence of phosphorylated JNK (Figures 5(a)–5(c)). We also observed an increase in Drp1 phosphorylation after H/R injury. Interestingly, supplementation of Mito-Q repressed JNK phosphorylation and inhibited Drp1 phosphorylation (Figures 5(a)–5(c)), suggesting that mitochondrial ROS promote JNK and Drp1 phosphorylation (p-JNK and p-Drp1) in CECs. This finding was also supported by our immunofluorescent findings. As shown in Figures 5(d)–5(f), the expressions of p-Drp1 and p-JNK were significantly increased in H/R-treated cells as compared with control cells. Interestingly, Mito-Q treatment repressed the increase in p-Drp1 and p-JNK following H/R injury (Figures 5(d)–5(f)). These findings suggest that mitochondrial fission in CECs is controlled by the ROS-Drp1-JNK signaling pathway.

## 4. Discussion

Coronary artery no-reflow is a complex problem in the area of reperfusion therapy [48]. Although there has been an advancement in drugs and interventional therapies in recent years, breakthrough progress has not been achieved in the prevention and treatment of coronary no-reflow injury following reperfusion therapy [2, 49]. Since infarction size, cardiac remodeling, myocardial function, and all-cause death are also determined by coronary no-reflow injury; understanding the molecular mechanisms underlying coronary no-reflow will promote specific diagnoses and therapies in clinical practice [11, 50]. In the present study, we used an H/R injury model to mimic coronary artery no-reflow injury *in vitro*. Our findings demonstrated that CEC damage was associated with mitochondrial dysfunction that was caused by increased mitochondrial fission. Further, we reported that oxidative stress was the primary inducer of mitochondrial fission via the activation of the JNK-Drp1 signaling pathway. ROS promoted JNK phosphorylation, which augmented Drp1 phosphorylation and resulted in mitochondrial fission in CECs. Excessive mitochondrial fission impaired mitochondrial bioenergetics, as evidenced by decreased ATP production and blunted mitochondrial respiration complex, which ultimately contributed to endothelial cell death. Overall, these findings demonstrate the molecular basis underlying ROS-induced endothelial cell damage and also identify the ROS-JNK-Drp1 pathway as the potential therapeutic target for the treatment of coronary no-reflow.

At the stage of myocardial ischemia, mitochondrial respiration is significantly impaired and glycolysis is augmented because of insufficient oxygen supply and interrupted blood flow, resulting in the accumulation of lactic acid [51, 52]. Since carbon dioxide in the blood stream cannot be quickly removed, the pH in endothelial cells significantly decreases [53]. As the pH decreases, massive hydrogen ions within endothelial cells promote the opening of Na^+^/H^+^ channels, which in turn promotes sodium translocation into the cytoplasm [54]. Subsequently, abnormal sodium stimulates sodium-calcium exchange channels that trigger intracellular calcium overload, which leads to coronary spasm and blunted coronary artery relaxation [55]. These alterations promote slow blood flow or terminal blood flow under hypoxic conditions. Although the recovery of blood flow will bring sufficient oxygen to the ischemic zone during the reperfusion stage [56], the damage to mitochondrial respiration that is induced by ischemic stress cannot be quickly restored in a short time, and this results in excessive ROS production in coronary endothelial cells [57]. In addition to oxidative stress, the inflammatory response is also initiated at the reperfusion stage. An uncontrolled inflammation response will aggravate myocardial edema [58] and ultimately compress the coronary artery and limit the diastolic function of the coronary artery. More importantly, inflammatory responses will also activate platelets to induce the formation of coronary thrombi [59, 60], leading to microcirculation embolism in the reperfusion phase. These alterations promote the development of coronary no-reflow injury even though the occluded epicardial arteries have been opened.

In the present study, we found that ROS are induced by H/R injury in CECs and correlate with the survival of CECs by mediating mitochondrial bioenergetics. Similarly, it was previously reported that ischemia/reperfusion-mediated senescence and vascular dysfunction of endothelial cells were attenuated by oxidants [61]. Endothelial cell necroptosis is also induced by ROS-mediated opening of the mitochondrial permeability transition pore (mPTP) [62]. Endothelial nitric oxide (NO) is a key regulator of vascular tone, and increased NO is associated with coronary relaxation. However, excessive oxidative stress impairs the bioactivity of NO and ultimately represses NO-mediated vascular relaxation [63]. Mitochondrial fragmentation is induced by superoxide anion production in coronary endothelial cells in diabetic mice [64], suggesting a possible relationship between mitochondrial fission and oxidative stress. In a mouse model of cardiac ischemia/reperfusion injury, mitochondrial fission in coronary endothelial cells was regulated by the nicotinamide adenine dinucleotide phosphate (NADPH) oxidase 2 (Nox-2) signaling pathway and ROS production [65]. This finding confirms that oxidative stress plays a causal role in mediating mitochondrial fission in endothelial cells. Our data further identified that the JNK-Drp1 signaling pathway was the primary mechanism responsible for ROS-modified mitochondrial fission in coronary endothelial cells. This finding will help us to better understand the relationship between oxidative stress and mitochondrial dynamics in coronary endothelial cells during coronary no-reflow injury.

Mitochondrial dynamics are mitochondrial morphological mechanisms that include mitochondrial fission and fusion. Mitochondrial fission is activated by hypoxia and/or reoxygenation, and multiple studies have confirmed that mitochondrial fission plays a pathological role in exacerbating myocardial damage during reperfusion therapy. For example, it was demonstrated that cardiac function was improved by the inhibition of mitochondrial fission at the reperfusion stage [66]. Another study revealed that reperfusion-mediated energetic crises were attenuated by mitochondrial fission suppression in a mouse model of cardiac ischemia/reperfusion injury [67]. It was also reported that intimal thickening after coronary damage was enhanced by mitochondrial fission due to dysregulated macrophage function [68, 69]. Further, it was demonstrated that inflammation and apoptosis of coronary endothelial cells were reduced via inhibition of mitochondrial fission or activation of mitophagy [70]. Decreased mitochondrial fission significantly promotes the production of NO in coronary endothelial cells, and this effect is followed by increased endothelium-dependent vasodilation [71]. Similarly, our data revealed that mitochondrial fission caused endothelial cell dysfunction as evidenced by decreased cell viability, impaired mitochondrial bioenergetics, and reduced ATP production. Based on these findings, mitochondrial fission may be a potential therapeutic target that sustains endothelial cell function and viability.

Overall, our results illustrate that coronary no-reflow injury is associated with endothelial cell damage that is caused by excessive oxidative stress. The ROS-JNK-Drp1 signaling pathway is activated at the stage of cardiac ischemia/reperfusion injury and contributes to mitochondrial fission, resulting in abnormal mitochondrial function and decreased endothelial cell viability. However, there are several limitations in the present study. First, our results are primarily based on cellular experiments, and animal studies are necessary to support our findings. Second, we used chemical inhibitors to determine the role of the ROS-JNK-Drp1 signaling pathway in regulating endothelial cell viability during coronary no-reflow. Additional studies using genetic ablation mice are required to confirm our findings.

## Data Availability

The analyzed datasets that were generated during the study are available from the corresponding author upon reasonable request.
